# Alemtuzumab as a Therapy for Chronic Lung Allograft Dysfunction in Lung Transplant Recipients With Short Telomeres

**DOI:** 10.3389/fimmu.2020.01063

**Published:** 2020-05-28

**Authors:** Anil J. Trindade, Tany Thaniyavarn, Keri Townsend, Robin Klasek, Karen P. Tsveybel, John C. Kennedy, Hilary J. Goldberg, Souheil El-Chemaly

**Affiliations:** ^1^Pulmonary and Critical Care Medicine, Brigham and Women's Hospital, Boston, MA, United States; ^2^Harvard Medical School, Boston, MA, United States; ^3^Division of Allergy, Pulmonary and Critical Care Medicine, Vanderbilt University Medical Center, Nashville, TN, United States

**Keywords:** alemtuzumab, chronic lung allograft dysfunction, lung transplantation, short telomeres, chronic rejection, telomere length, augmented immunosuppression

## Abstract

Alemtuzumab, a monoclonal antibody targeting CD52 that causes lymphocyte apoptosis, is a form of advanced immunosuppression that is currently used as a therapy for refractory acute cellular rejection and chronic lung allograft dysfunction in lung transplant recipients (1–3). Side effects of alemtuzumab include bone marrow suppression, infection, and malignancy. Whether alemtuzumab can be safely used in allograft recipients that have an increased propensity for bone marrow suppression due to telomeropathies is unknown. In a retrospective case series, we report outcomes associated with alemtuzumab in three lung allograft recipients with short telomere lengths, comparing endpoints such as leukopenia, transfusion needs, infection, hospitalization and survival to those of 17 patients without known telomeropathies that received alemtuzumab. We show that the use of alemtuzumab in lung transplant recipients with short telomeres is safe, though is associated with an increased incidence of neutropenia, thrombocytopenia and anemia requiring packed red blood cell transfusions. Alemtuzumab appears to be an acceptable advanced immunosuppressive therapy in patients with telomeropathies, though given the design and scope of this study, the actual clinical effect needs further evaluation in larger trials.

## Introduction

Lung transplantation is a life-saving therapy for patients with end-stage lung disease recalcitrant to medical therapies (4). While lung transplantation is associated with improved survival and quality of life, outcomes are limited by the development of chronic lung allograft dysfunction (CLAD) (4–7). Over 50% of lung transplant recipients will develop CLAD by 5 years post-transplant (8–10). Therapies for CLAD are limited. The macrolide antibiotic, azithromycin, is generally used as a first-line agent to minimize inflammation, particularly in the neutrophilic reversible allograft dysfunction phenotype (11, 12). Montelukast, a leukotriene inhibitor, may also help stabilize lung function in patients with a more mild stage of CLAD (13–15). Small studies have shown the potential for advanced immunosuppressive therapies such as extracorporeal photopheresis or alemtuzumab, for the treatment of refractory acute cellular rejection (ACR) and severe CLAD (2). Alemtuzumab is a monoclonal antibody that binds to CD52 on lymphocytes and promotes a sustained apoptotic response, often leading to undetectable levels of CD3/CD4 lymphocytes for years (16). In retrospective studies, alemtuzumab has been shown to be efficacious in stabilizing lung function (2, 3, 17). However, the potential for sustained bone marrow suppression seen with alemtuzumab is of concern, given the susceptibility of lung transplant recipients to infections and malignancies; this concern may be more pronounced in patients with predispositions to bone marrow suppression, including those with short telomeres.

Telomeres are non-coding hexanucleotide tandem repeats at the ends of chromosomes that protect them from gradual degradation. Patients with telomeropathies are at risk for chromosomal damage and can ultimately develop bone marrow suppression, skin abnormalities, hepatic dysfunction, pulmonary fibrosis, and malignancies (18–20). Moreover, patients with inherited forms of pulmonary fibrosis (including those due to telomeropathies) are known to have both abnormal quantities of immune mediator cells and altered immune cell function (21, 22). Detection of short telomere lengths in patients with end-stage lung disease requiring lung transplantation is not uncommon, given the predisposition to pulmonary fibrosis in these patients (18, 23). In the Brigham and Women's cohort, about 15% of lung transplant recipients with interstitial lung disease have been found to have short telomere lengths, although this likely under-represents the true prevalence, given that a pragmatic approach to telomere length screening was utilized, assaying only individuals with disease manifestations (18). Unfortunately, lung transplant recipients with short telomeres have worse outcomes, including decreased CLAD-free survival and overall survival (24, 25). They may also have other co-morbidities post-transplant including bone marrow-suppression, intolerance of immunosuppression and increased rates of renal dysfunction (24).

We recently counseled a patient with short telomere syndrome who was ~2-years status-post bilateral lung transplant on treatment options for rapidly progressive CLAD. During the discussion it became evident that there is a deficit of literature regarding the effects of alemtuzumab on patients with short telomeres. We wondered if lung transplant recipients with short telomere lengths experience profound bone marrow suppression with alemtuzumab, and whether that might be associated with increased morbidity including cytopenias, infections, increased hospitalizations and untimely death. We performed a retrospective analysis of our cohort to determine if alemtuzumab in patients with short telomeres is associated with acceptable outcomes and report the results as a case series of 3 individuals. Outcomes are compared to 17 lung transplant recipients without known telomeropathies that received alemtuzumab.

## Methods

Permission to perform this retrospective analysis was obtained from the Partners institutional review board (IRB#2018P002267). Lung allograft recipients transplanted at BWH between 1/1/2012 - 12/31/2018 and who received alemtuzumab for refractory ACR or CLAD were included in this retrospective analysis. Patient outcomes were assessed through 11/12/2019. ACR was diagnosed in accordance with International Society of Heart and Lung Transplant guidelines (26). ACR was defined as refractory if there was a significant decline in spirometry (>10% change from peak FEV1) despite corticosteroid pulse and taper (27). Notably, our center began to routinely assess for telomere lengths in the pre-transplant setting in high-risk patients in 2012 (18). Patients underwent telomere length testing if they met any of the following criteria: family history of interstitial lung disease, premature graying, evidence of bone marrow dysfunction and/or evidence of hepatic dysfunction. Telomere length testing was performed using combined flow cytometry and FISH, which enables telomere length analysis in six different cell lines (Repeat Dx, North Vancouver, BC, Canada). Results are age-adjusted. Telomere length was defined as “Low” if it fell below the tenth percentile of a reference range of healthy controls and “Very Low” if it was below the first percentile. In this analysis patients were classified as having short telomeres if lymphocyte telomere length was less than the 10th percentile corrected for age (18, 28). Patients with suspected or documented short telomeres also underwent bone marrow biopsy as part of the lung transplant evaluation process.

Lung transplant recipients at BWH were managed per usual standard of care, as previously described, which includes induction therapy [antithymocyte globulin (Thymoglobulin®) or anti-IL-2R (basiliximab)] and three-drug immunosuppression with a calcineurin inhibitor (preferably tacrolimus), an anti-proliferative agent (preferably mycophenolate mofetil) and a corticosteroid (prednisone) (2). Patients received CMV or HSV prophylaxis for 6-12 months depending on donor and recipient serologic status. Patients also received Pneumocystis jirovecii (PJP) prophylaxis lifelong (trimethoprim/sulfamethoxazole preferred). Upon administration of alemtuzumab, patients had their anti-proliferative immunosuppressant discontinued. Moreover, prophylaxis against CMV and mold (using voriconazole or isavuconazole) was instituted until the bone marrow recovered (as evidenced by CD4+ lymphocyte counts > 200/uL). If patients developed persistent cytopenias then trimethoprim/sulfamethoxazole prophylaxis was changed to atovaquone in patients that were Toxoplasma IgG negative (donor and recipient and HSV/CMV prophylaxis was withheld and patients were monitored weekly for recrudescent or *de novo* viremia.

Patients were included in our analysis if they survived > 3 months post-administration of alemtuzumab.

### Definitions

We assessed for several different outcomes post-administration of alemtuzumab; any complication occurring more than 7 days post- administration of alemtuzumab was included. Outcomes assessed include leukopenia (total WBC <4,000/uL), neutropenia (ANC <1000/uL), lymphocytopenia (ALC <1000/uL), thrombocytopenia (platelets <150,000/uL), need for packed red blood cells (PRBCs), platelets, or granulocyte colony stimulating factor (G-CSF), time to CD4+ lymphocyte recovery (>200 cells/mL), hospital readmission, infection requiring hospitalization, occurrence of malignancy, CMV viremia (>137 copies of CMV DNA in serum), EBV viremia (>2,000 copies DNA) and time to death. At BWH, G-CSF is routinely given if absolute neutrophil counts are <1000 despite adjustment of bone marrow-suppressive medications, regardless of presence of infection. Hospital readmission was defined as any unplanned hospitalization. Infection was defined as any suspected or documented organ dysfunction due to a microorganism that required hospitalization, and for which antimicrobials were prescribed.

### Statistical Analysis

Statistical analysis was performed using STATA version 15. 1 (StatCorp LLC, College Station, TX). For all results, *p* ≤ 0.05 were considered significant. Differences in baseline demographic data were assessed using Fisher's Exact test for binary data. We performed univariate analyses using Fisher's Exact test to assess for significant differences between alemtuzumab and telomere length for binary outcomes.

## Results

Twenty-two patients who underwent lung transplantation between 1/1/2012 and 12/31/2018 ultimately received alemtuzumab for either refractory ACR or CLAD. Of those patients, 2 died within 90 days of alemtuzumab administration and were excluded from the analysis; these patients did not have known telomeropathies. Of the remaining 20 patients, 4 patients met pre-specified criteria to undergo telomere length testing (see criteria listed in the Methods section). Three of the four patients who were tested met criteria for having short telomere lengths, with documented lymphocyte telomere lengths <10th percentile. See Table 1 for further details. The other 17 patients did not meet our pre-specified criteria to undergo telomere length analysis. Notably, while all three patients had low lymphocyte telomere lengths, patient #1 had very low telomere lengths in the lymphocyte lineage, with age-matched lengths <1st percentile. Pre-transplant bone marrow biopsy results mirrored the degree of involvement of telomeropathies (See Table 1); patient #1 had markedly low cellularity, while patients #2 and #3 had moderately reduced cellularity.

**Table 1:** Age-adjusted telomere lengths in various cell lines and bone marrow biopsy results in patients with short telomeres.

**Patient**	**Telomere Lengths**						**Bone Marrow Biopsy Results**
	**Lymphocytes**	**Granulocytes**	**CD45 RA+ Naïve Cells**	**CD45 RA+ Memory Cells**	**CD20+ B cells**	**CD57+ NK Cells**	
1	VL	VL	VL	L	VL	L	Markedly hypocellular marrow with trilineage hematopoiesis with left shifted myeloid elements.
2	L	VL	L	L	L	N	Moderately hypocellular with maturing trilineage hematopoiesis
3	L	VL	L	L	L	N	Moderately hypocellular with maturing trilineage hematopoiesis

*Normal (N) ≥ 10th percentile*.

*Low (L) ≤ 10th percentile but > 1st percentile*.

*Very Low (VL) ≤ 1st percentile*.

Baseline characteristics for patients receiving alemtuzumab are shown in Table 2. In our small cohort, there were no significant differences in baseline characteristics of patients with short telomeres vs. those patients without known telomeropathies who received alemtuzumab for ACR or CLAD.

**Table 2:** Baseline characteristics of patients that received alemtuzumab for ACR or CLAD.

	**Short Telomere Length (*****N*** **=** **3)**			**Without Known Telomeropathy (*N* = 17)**	***P*=**
	**#1**	**#2**	**#3**		
Age > 60 years at transplant	No	Yes	Yes	9 (56%)	1.000
Female Sex	No	Yes	Yes	7 (41%)	0.566
Bilateral lung transplant	Yes	Yes	Yes	11 (65%)	0.521
CMV D+/R- Status	No	Yes	Yes	5/16 (31%)	0.523
EBV D+/R- Status	No	No	No	1 (6%)	1.000
**Type of end-stage lung disease**					
Obstructive	–	–	–	4 (24%)	0.539
Pulmonary vascular	–	–	–	1 (6%)	
Cystic fibrosis	–	–	–	0	
Interstitial	Yes	Yes	Yes	12 (71%)	
Chronic lung allograft dysfunction as indication for alemtuzumab	Yes	Yes	Yes	11 (69%)	0.521
Bronchiolitis obliterans syndrome	Yes	Yes	Yes	10/11 (91%)	1.000
Restrictive allograft syndrome	No	No	No	1/11 (9%)	1.000
Alemtuzumab administered <2 years post-transplant	No	Yes	Yes	10 (59%)	0.546

**Figure 1:**
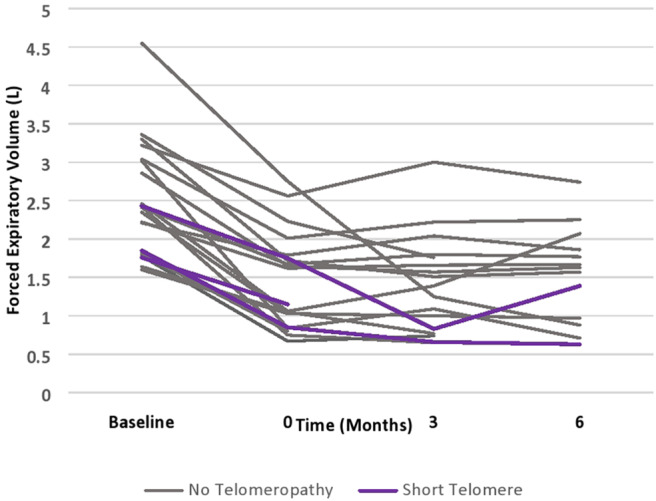
Allograft function in patients after receiving Alemtuzumab.

Patients with short telomeres who received alemtuzumab had significantly increased incidence of neutropenia (66 vs. 5.9%, *p* = 0.046), thrombocytopenia (100 vs. 23.5%, *p* = 0.031), and anemia requiring PRBCs (66 vs. 5.9%, *p* = 0.046). There was no significant difference in unplanned hospitalizations, infections necessitating hospitalization, lymphocytopenia, need for G-CSF therapy or CMV or EBV viremia. Moreover, there did not appear to be numerical differences in post-alemtuzumab survival, though this could not be statistically analyzed (Table 3). There did appear to be a trend towards greater response to alemtuzumab in patients without known telomeropathy, with greater stability of FEV1 over a 6-month period following therapy administration, though the small sample size precludes statistical analysis (Figure 1).

**Table 3:** Outcomes in patients receiving Alemtuzumab.

	**Short telomere length (*****N*** **=** **3)**			**Without known telomeropathy (*N*= 17)**	**P=**
	**#1**	**#2**	**#3**		
Unplanned hospitalizations	Yes	Yes	Yes	14 (82.3%)	1.000
Infection requiring hospitalization	No	Yes	No	3 (17.6%)	0.509
Malignancy	No	No	No	1 (5.9%)	1.000
Leukopenia	Yes	Yes	Yes	16 (94.1%)	1.000
Lymphocytopenia (<1000/ul)	Yes	Yes	Yes	16 (94.1%)	1.000
Neutropenia (anc <1000)	Yes	Yes	No	1 (5.9%)	**0.046**
G-CSF therapy	Yes	Yes	No	4 (23.5%)	0.202
Thrombocytopenia	Yes	Yes	Yes	4 (23.5%)	**0.031**
Prbc transfusions	Yes	Yes	No	1 (5.9%)	**0.046**
Platelet transfusions	Yes	No	No	0	0.150
Cd4 <200/ml	
Died before recovery	Yes	–	–	7 (41.1%)	1.000
Alive without recovery	–	Yes	Yes	4 (23.5%)	0.202
Time to CD4 >200/ml (days)^**^	–	–	–	778 (605–962)	N/A
CMV viremia	Yes	No	No	1 (5.9%)	0.284
EBV viremia	No	No	No	0	1.000
Post-alemtuzumab survival (days)^*^	471	620+	100+	368 (175–1040)	N/A
Delta fev1 (l) post-alemtuzumab^***^	−0.22	−0.36	N/A	0.05 (-0.09–0.14)	N/A
Delta fvc (l) post-alemtuzumab^***^	−0.18	−0.92	N/A	0.11 (-0.16–0.33)	N/A

* *At the time of this analysis 14/33 (35%) patients are still alive*.

** *4 patients had recovery of CD4 T lymphocytes following treatment with alemtuzumab*.

*** *Patient #3 was too unstable to have spirometry measured following alemtuzumab therapy. Bolded values are ones that are significant (p < 0.05)*.

## Discussion

To our knowledge, this is the first report of the use of alemtuzumab in lung transplant recipients with short telomeres. In our small case series of 3 patients, we find that alemtuzumab in patients with short telomeres is associated with increased incidence of cytopenias, including neutropenia, thrombocytopenia, and anemia requiring PRBC transfusions. The episodes of thrombocytopenia were not associated with an increased need for platelet transfusions. Similarly, prevalence of neutropenia was not associated with a significant trend towards greater use of G-CSF. There was no significant effect of cytopenias on rates of infection or hospitalization. While alemtuzumab appeared to stabilize lung function in patients without known telomeropathy, its effect did not appear to be as potent in patients with short telomeres, though the sample size was too small to make definitive conclusions. Notably, the rate of decline of FEV1 in patients with CLAD that do not receive advanced immunosuppressive therapy has been shown to be ~0.1 L/month, so patients with short telomeres do seem to derive some benefit, albeit a muted one (2).

We are careful to not overstate findings from this series, given the limited number of patients that met inclusion criteria. The number of lung allograft recipients with underlying short telomeres that were treated with alemtuzumab for refractory ACR or CLAD in our cohort is limited. Another limitation of this study is that telomere length analysis was not performed in patients that did not meet specified criteria; the possibility that these patients do not have short telomeres cannot be definitively excluded. This could potentially mute outcomes between groups.

Despite the limitations of our study, these findings are relevant given the increasing identification of short telomere syndromes in lung transplant recipients and the high rate of CLAD in lung allograft recipients. While there are currently no FDA-approved therapies for CLAD, there is evidence that alemtuzumab as advanced immunosuppression is associated with improved stability in lung function (1, 2, 17). Therefore, it is likely that lung transplant physicians will increasingly be faced with the dilemma of treating CLAD with bone marrow suppressive therapies such as alemtuzumab in patients with short telomeres.

Short telomeres are associated with a variety of hematologic effects with different degrees of severity (29). Recent evidence suggests that T cells from patients with short telomere syndrome show stigmata of a premature aging phenotype, and a distinct molecular program with upregulated intrinsic apoptosis and DNA-damage double-strand break response (22). While the number of patients with short telomeres receiving alemtuzumab in our cohort is too small to make definitive conclusions, our data suggests that alemtuzumab exacerbates the propensity for bone marrow disarray, with increased incidence of cytopenias. However, it is reassuring that patients with short telomeres in our cohort who received alemtuzumab did not seem at higher risk of infection, albeit in the presence of appropriate anti-fungal, viral and PJP prophylaxis. Certainly, patients with telomeropathies receiving alemtuzumab do warrant close monitoring.

As more reports are published about the safety of lung transplantation in patients with short telomeres, and since short telomeres are associated with shorter time for development of CLAD (30), it is important that therapeutic options for this patient population are investigated. Our report is the first to offer insight into the use of alemtuzumab in patients with short telomeres; based on our findings alemtuzumab seems to be an acceptable therapeutic option in this patient population, albeit associated with increased side effects. Certainly, well designed prospective studies assessing outcomes of the management of lung transplant recipients with short telomeres are needed.

## Data Availability Statement

All datasets generated for this study are included in the article/supplementary material.

## Ethics Statement

The studies involving human participants were reviewed and approved by Brigham and Women's Institutional Review Board. Written informed consent for participation was not required for this study in accordance with the national legislation and the institutional requirements.

## Author Contributions

AT and SE-C contributed conception and design of the study. AT, KTo, RK, and TT organized the database. AT performed the statistical analysis. AT and SE-C wrote the first draft of the manuscript. All authors contributed to manuscript revision and read and approved the submitted version.

## Conflict of Interest

The authors declare that the research was conducted in the absence of any commercial or financial relationships that could be construed as a potential conflict of interest.

## Abbreviations

ACR: Acute cellular rejection
ALC: Absolute lymphocyte count
ANC: Absolute neutrophil count
BWH: Brigham and Women's Hospital
CD: Cluster of differentiation/Classification determinant
CLAD: Chronic lung allograft dysfunction
CMV: Cytomegalovirus
EBV: Epstein Barr Virus
FEV1: Forced expiratory volume in 1 second
FISH: Fluorescent *in-situ* hybridization
G-CSF: Granulocyte colony stimulating factor
HSV: Herpes simplex virus
IQR: Interquartile range
NK: Natural Killer.
